# Data in support for the measurement of serum 25-hydroxyvitamin D (25OHD) by tandem mass spectrometry

**DOI:** 10.1016/j.dib.2016.07.017

**Published:** 2016-07-15

**Authors:** M.E. Jensen, F.M. Ducharme, Y. Théorêt, A.-S. Bélanger, E. Delvin

**Affiliations:** a Centre for Asthma and Respiratory Diseases, School of Biomedical Sciences & School of Medicine and Public Health, University of Newcastle, Callaghan, Australia; b Departments of Pediatrics and Social and Preventive Medicine, University of Montreal, Montreal, Canada; c Clinical Research and Knowledge Transfer Unit, Research Centre, CHU Ste-Justine, Montreal, Canada; d Clinical Pharmacology Unit, Department of Clinical Biochemistry, CHU Ste-Justine, Canada; e Department of Pharmacology, University of Montreal, Montreal, Canada; f Department of Clinical Biochemistry, CHU Ste-Justine, Canada; g Gatroenterology, Hepatology & Nutrition Division, CHU Ste-Justine Research Centre, University of Montreal, Montreal, Canada

**Keywords:** Vitamin D, 25-hydroxyvitamin D, Mass spectrometry

## Abstract

This article provides data and a method related to a research paper entitled “Assessing vitamin D nutritional status: is capillary blood adequate?” (Jensen et al., 2016) [1]. Circulating 25OHD, the accepted biomarker of the vitamin D nutritional status, is routinely measured by automated immunoassays, that although may be performed in hospital central laboratories, often suffer from a lack of specificity with regards to the different vitamin D metabolites, “Measurement of circulating 25-hydroxyvitamin D: a historical review” (Le Goff et al., 2015) [2]. Mass spectrometry offers this specificity. This article describes the performance of an in-house tandem mass spectrometry method for the individual measurement of 25OHD_3_, 25OHD_2_ and 3-épi-25OHD_3_.

## Specifications Table

**Table:** 

Subject area	Laboratory medicine
More specific subject area	Clinical Chemistry
Type of data	Tables, figures
How data was acquired	Mass spectrometry, Agilent 6460 triple quadrupole mass spectrometer equipped with a JetStream™ interface coupled to
Data format	Mass spectral analysis
Experimental factors	Charcoal-stripped serum served as a blank. Deuterated [25OHD_3_ (26,26,26,27,27,27-d_6_, IS_1_), 25OHD_2_ (26,26,26,27,27,27-d_6_, IS_2_) and 3-epi-25OHD_3_ (6,19,19-d_3_, IS_3_) served as internal standards for each vitamin D metabolite quantitation.
Experimental features	The mass spectral analysis was performed using a MassHunter workstation software, version B.04.00 (Agilent Technologies Canada Inc., Mississauga, ON, Canada).
Data source location	Montreal, Québec, Canada
Data accessibility	The data is available with this article

## Value of the data

• The data describes a tandem mass spectrometry method for the measurement of serum 25OHD.
• The details given enable other researchers to reproduce this method.
• These data will be useful for implementing tandem mass spectrometric methods for the quantification of vitamin D metabolites in future clinical studies.

## 1 Data

Data Capillary blood has been shown to be an adequate matrix for measuring 25OHD [1[. Automated immunoassays routinely used by hospital central laboratories suffer from a lack of specificity that mass spectrometry offers [2].

The data shared include the description of the extraction, chromatography and mass spectrometry protocols as well sample mass spectra obtained from standards and extracted serum. The validation procedure of the method and results are also described.

## 2 Experimental design, materials and methods

100 μL of a blank, consisting of charcoal-stripped plasma (cat. #1131-00) purchased from Biocell (Rancho Dominguez, CA, USA), sample, calibrator or control were transferred to glass tubes, and spiked with 250 μL of a mixture of deuterated internal standards 25OHD_3_ (26,26,26,27,27,27-d_6_, IS_1_), 25OHD_2_ (26,26,26,27,27,27-d_6_, IS_2_) from Chemaphor Inc., (Ottawa, ON, Can) and 3-epi-25OHD_3_ (6,19,19-d_3_, IS_3_) from Sigma-Aldrich Canada (Oakville, ON, Can) mixed gently for 10 s, incubated for 1 h at room temperature with periodic short mixing, before the addition of 1.0 mL of 2-methoxy-2-methylpropane/hexane (50/50 v/v). After a further 10-min incubation at room temperature, the mixture was centrifuged at 3000 RPM for 5 min. 800 μL of the supernatant was transferred to a 12/75-glass tube, evaporated to dryness at 37 °C under a stream of N_2_. The crude extract was reconstituted in 100 μL of the initial HPLC mobile phase (see below) and placed on the auto-sampler.

**Fig. 1:**
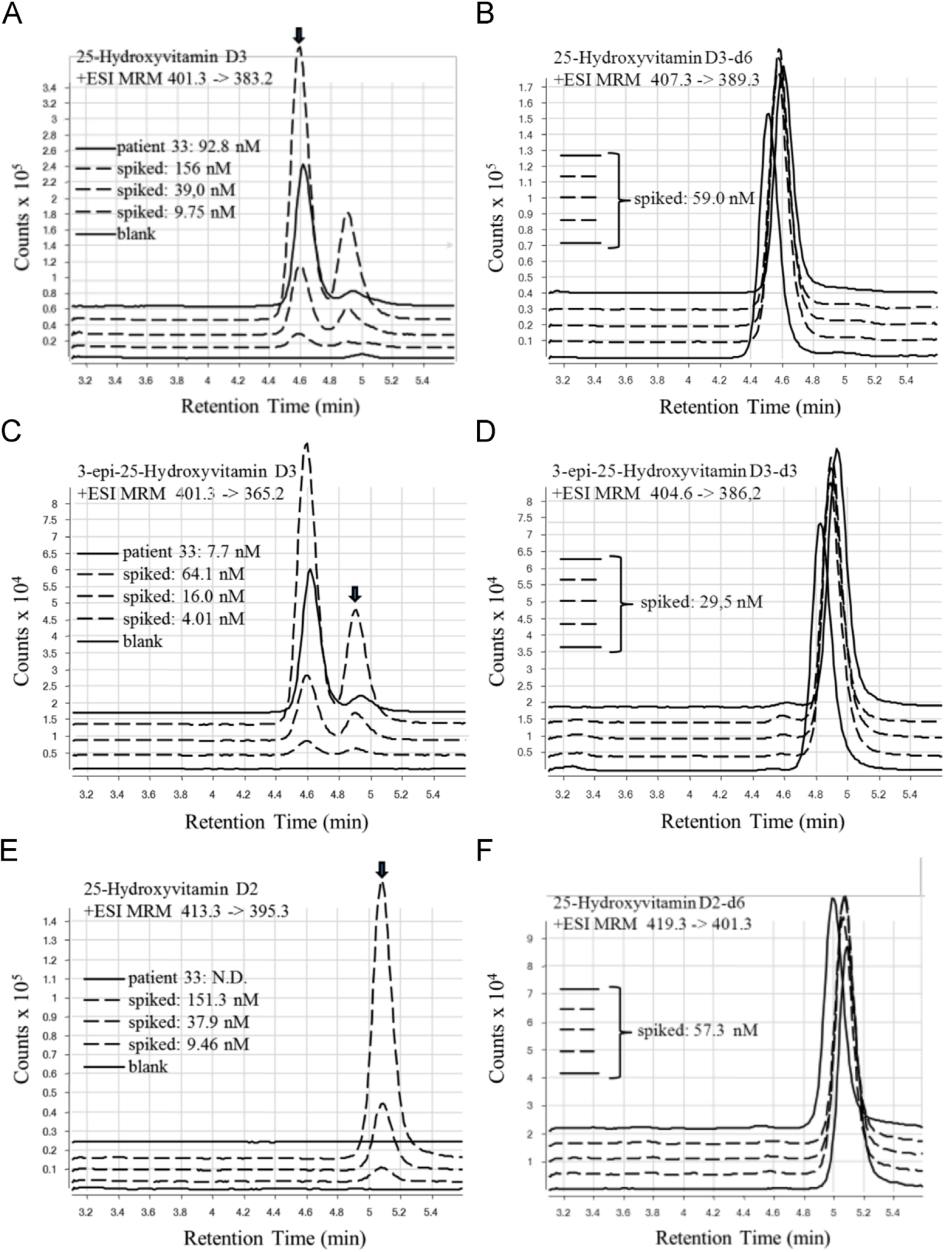
Representative selected ion LC-MS/MS chromatograms of charcoal-stripped blank plasma (lower solid line) and charcoal-stripped blank plasma spiked with vitamin D metabolites (dashed lines), and a patient plasma (upper solid line) using a PentaFluoroPhenyl Core shell Silica column. Panels A, C and E: Arrows indicate 25OHD_3_, 3-epi-25OHD_3_, 25OHD_2_ respectively. All plasma samples were spiked with deuterated internal standards (ion chromatograms shown in panels B, D and F). Deuterated and non-deuterated compounds had same retention times.

The detailed chromatography and optimized+ion mode ESI-MS/MS conditions and ionic transition masses used are described in Table 1. HPLC/ESI MS-MS (high performance liquid chromatography/electrospray ionization tandem mass spectrometry) was performed on an Agilent 1200 HPLC system, consisting of degassers, binary and quaternary pumps, an auto-sampler equipped with a micro Rheodyne valve, and a temperature controlled column compartment with a column-switching valve coupled to an Agilent 6460 triple quadrupole mass spectrometer equipped with a JetStream™ interface (Agilent Technologies Canada Inc., Mississauga, ON, Canada). For the chromatography, 10 μl of the extracts were injected on a jacket-heated (50 °C) column [100×2.1 mm Kinetex™ 2.6 µm PentaFluoroPhenyl Core shell Silica 100 Å (Phenomenex, Torrance, CA, USA)] preceded by a 2.1 mm internal diameter (ID) SecurityGuard™ ULTRA cartridge (Phenomenex). The MassHunter workstation software, version B.04.00 (Agilent Technologies) was used for the management of the HPLC/ESI MS-MS system and data acquisition. The spectral analysis was done in the Multiple Reaction Mode (MRM). Fig. 1 illustrates representative selected ion LC-MS/MS chromatographic profiles of spiked charcoal-stripped plasma spiked with vitamin D standards and deuterated internal standards, and a patient serum extract analyzed in the conditions described above.

### 2.1 Analytical performance

The method validation was performed with modified CLSI Guidelines [3], [4]. Briefly, the Lower Limit of quantification (LLoQ) was estimated by the serial dilution of the standard solution (*n*=5 per dilution) and was defined as the concentration at which precision was **≤**20%. Linearity was evaluated by serially diluting a pool of high 25OHD_3_ concentration samples with charcoal-stripped serum to generate 8 samples of intermediate concentrations that were measured in duplicate.

The MassCheck® controls for the 3 analytes (Chromsystems Instruments and Chemicals GmbH, Gräfelfing, Germany) traceable to NIST 972a standard reference material, served for precision and bias assessment. For within-assay imprecision, 5 sequential injections of each concentration of the MassCheck® controls were performed. Between-assay imprecision was assessed by analyzing 1 reference sample at 2 different concentrations in each batch over 14 months.

Linear responses were observed up to 312 nmol/L for 25OHD_3_, and 25OHD_2_ and 128 nmol/L for 3-epi-25OHD_3_. Table 2 summarizes the performance characteristics of the method. The LLOQ for 25OHD_3_, 25OHD_2_ and 3-epi-25OHD_3_ were 7.0 nmol/L, 5.0 nmol/L and 4.0 nmol/L, respectively. The intra- and inter-assay imprecisions were respectively ≤2.5% and ≤6.0% for all metabolite measured. The mean bias for 25OHD_3_ was 1.4% and 4.8% at 37.9 and 87.5 nmol/L respectively, within the limits set by the Vitamin D Standardization Program [5].

## Figures and Tables

**Table 1:** Analytical data.

***Chromatography***				
The mobile phases were: (A) H_2_O/methanol (50/50 v/v)+0.1% formic acid, and (B) methanol+0.1% formic acid. The needle rinse solvent was acetonitrile/methanol/2-propanol (50/25/25). The binary pump flow rate was set at 0.4 mL/min with 44% B for the first 6 min (flow was switched away from cartridge at 3 min for a backwash step with the quaternary pump) followed by 100% B for a 1 min wash step before returning to 44% B for at least a 2 min post-run. The quaternary pump flow rate was set at 0.4 mL/min with 100% B from 0 to 6 min (the flow was directed to waste for the first 3 min and in a back-flush mode for the cartridge in the last 3 min) followed by 44% B for 1 min and 100% B for at least a 2 min post-run.				
**Mass spectrometer settings**				
The optimized+ion mode ESI-MS/MS conditions were as follows: gas temperature 275 °C, gas flow 5 L/min, sheath gas heater 325 °C, sheath gas flow 11 L/min: nebulizer 45 psi, and capillary voltage 5000 V. Nitrogen was used as desolvation and collision gas. The multiplier voltage was set at 0 V except between 2.9 and 6 min where it was set at 635 V, the interval during which the flow is directed to the nebulizer and not diverted to waste.				



Vitamin D metabolite	Transition ion m/z	Dwell time (ms)	Fragmentor (V)	CE (V)
25OHD_3_	401.3→383.2	100	106	35
[^6^d_2_]-25OHD_3_	407.3→389.3	100	106	35
25OHD_2_	413.3→395.3	100	100	32
[^6^d_2_]-25OHD_2_	419.3→401.3	100	106	32
3-epi-25OHD_3_	401.3→365.2	100	106	35
[^3^d_2_]-3-epi-25OHD_3_	404.6→386.2	100	106	35

**Table 2:** Method performance.

Vitamin D metabolite	LLoQ nmol/L	Intra-assay CV% (nmol/L)^a^	Inter-assay CV% (nmol/L)^b^	Bias % (nmol/L)^b^
25OHD_3_	7.0	3.34 (40.6)	5.3 (38.4)	1.4 (37.9)
		0.79 (95.2)	4.3 (91.7)	4.8 (87.5)
				
25OHD_2_	5.0	4.02 (40.4)	5.9 (46.8)	11.2 (42.1)
		1.09 (93.6)	3.8 (113.1)	4.7 (108.0)
				
3-epi-25OHD_3_	4.0	2.07 (36.2)	6.0 (39.3)	14.9 (34.2)
		2.32 (61.0)	3.7 (67.5)	23.8 (54.5)

LLoQ: Lower limit of quantification.

a 5 sequential injections of each concentration of the MassCheck® controls were performed. Values in parentheses are the means.

b 1 aliquot of each concentration of the MassCheck® controls was analyzed over a period of 14 consecutive months [*n*=90]. For the inter-assay repeatability, values in parentheses are those obtained by our method. For the bias, values in parentheses are those assigned by the manufacturer.
